# Glyceraldehyde-3-phosphate dehydrogenase present in extracellular vesicles from *Leishmania major* suppresses host TNF-alpha expression

**DOI:** 10.1016/j.jbc.2021.101198

**Published:** 2021-09-15

**Authors:** Priya Das, Aditi Mukherjee, Subrata Adak

**Affiliations:** Division of Structural Biology & Bio-informatics, CSIR-Indian Institute of Chemical Biology, Kolkata, India

**Keywords:** glyceraldehyde-3-phosphate dehydrogenase, extracellular vesicles, host–parasite interaction, TNF-α, *Leishmania*, AFM, atomic force microscopy, BSA, bovine serum albumin, CM, complement cell, CT, control cell, DLS, dynamic light scattering, EV, extracellular vesicle, FBS, fetal bovine serum, HKO, LmGAPDH half knockout cell, LmGAPDH, glyceraldehyde-3-phosphate dehydrogenase from *Leishmania major*, LPS, lipopolysaccharide, OE, LmGAPDH overexpressing cell, TNF-α, Tumor necrosis factor alpha, REMSA, RNA electrophoretic mobility shift assay

## Abstract

Glyceraldehyde-3-phosphate dehydrogenase (GAPDH) fulfills various physiological roles that are unrelated to its glycolytic function. However, to date, the nonglycolytic function of GAPDH in trypanosomal parasites is absent from the literature. Exosomes secreted from *Leishmania*, like entire parasites, were found to have a significant impact on macrophage cell signaling and function, indicating cross talk with the host immune system. In this study, we demonstrate that the *Leishmania* GAPDH (LmGAPDH) protein is highly enriched within the extracellular vesicles (EVs) secreted during infection. To understand the function of LmGAPDH in EVs, we generated control, overexpressed, half-knockout (HKO), and complement cell lines. HKO cells displayed lower virulence compared with control cells when macrophages and BALB/c mice were infected with them, implying a crucial role for LmGAPDH in *Leishmania* infection and disease progression. Furthermore, upon infection of macrophages with HKO mutant *Leishmania* and its EVs, despite no differences in *TNFA* mRNA expression, there was a considerable increase in TNF-α protein expression compared with control, overexpressed, and complement parasites as determined by ELISA, RT-PCR, and immunoblot data. *In vitro* protein translation studies suggest that LmGAPDH-mediated TNF-α suppression occurs in a concentration-dependent manner. Moreover, mRNA binding assays also verified that LmGAPDH binds to the AU-rich 3′-UTR region of *TNFA* mRNA, limiting its production. Together, these findings confirmed that the LmGAPDH contained in EVs inhibits TNF-α expression in macrophages during infection *via* posttranscriptional repression.

*Leishmania* spp., the organism causing leishmaniasis in humans, divides its life cycle between the sand fly vector and the mammalian host. Earlier researchers revealed that extracellular vesicles (EVs)-based secretion by *Leishmania* is involved in the delivery of proteins into host cells (1, 2). Comparative quantitative proteomics studies unambiguously identified numerous proteins in *Leishmania* EVs (1, 3). *Leishmania* EVs and EV mediate proteins were detected in the cytosolic compartment of infected macrophages, and the incubation of macrophages with EVs selectively regulated the secretion of different cytokines (2, 4). However, the exact functions of parasite-derived EV mediate proteins in host parasite interaction are far from being elucidated.

Earlier, glyceraldehyde-3-phosphate dehydrogenase (GAPDH) has been considered simply as a housekeeping glycolytic enzyme. Recent studies indicate GAPDH as a multifunctional protein displaying numerous physiological roles that are unrelated to its glycolytic function. For example, GAPDH shows phosphotransferase/kinase activity, autophosphorylation, or phosphorylation of other proteins, thus acting as a cellular kinase (5). It can interact with tubulin and catalyzes tubulin polymerization into microtubules (6), assists membrane fusion in a highly plasmenylethanolamine- and cholesterol-specific manner (7) and shows Ca^2+^-dependent fusogen activity (8). It has been identified as a target protein for nitric oxide (9) and a binding protein for nucleic acids, DNA (10), and nuclear tRNA (11). It has also been identified as a uracil DNA glycosylase (12) and as an Ap4A-binding protein (13), implying that it is involved in DNA replication and repair. GAPDH also acts as a specific mRNA-binding protein interacting with 5′-UTR or 3′-UTR mRNA sequences that are important for translational regulation of gene expression (14). Other known activities of GAPDH are the regulation of heme insertion in hemoproteins and nitrosylation of nuclear proteins (15, 16, 17). Furthermore, new and novel studies also indicate that moonlighting GAPDH has a fundamental role in a variety of pathologies including diabetes, age-related neurodegenerative disorders, and tumorigenesis (18, 19).

In some pathogens, GAPDH has been designated as a protein virulence factor (20, 21) owing to its ability to interact with several host proteins (22). Like *Trypanosoma brucei*, two copies of glycosomal GAPDH genes in tandem array and one copy of the cytosolic GAPDH genes have been identified in *Leishmania infantum*, *Leishmania mexicana*, and *Leishmania donovani*. The activity measurement data suggest that GAPDH in *T. brucei* and *L. mexicana* has also been detected in the cytosol (23, 24, 25). However, *Leishmania major* and *Leishmania braziliensis* have only two copies of the active form of glycosomal GAPDH and lack the cytosolic GAPDH genes (26, 27). A group of workers demonstrated that the cytosolic GAPDH enzyme in *L. donovani* plays a role in the parasite's ability to thrive in visceral organs (28). Although, GAPDHs from *T. brucei*, *Trypanosoma cruzi*, and *L. mexicana* have been purified, crystalized, and biochemically characterized (29, 30, 31), nonglycolytic role of GAPDH is still unknown in the trypanosomatid parasites, *Trypanosome* and *Leishmania*.

In this article, we have unraveled a possible nonglycolytic role of GAPDH from *L. major* (LmGAPDH), which is localized in the glycosome as well as in the EVs. To understand the nonglycolytic function of this protein, we have made different types of *Leishmania* cell lines (overexpressed [OE], control [CT], half knockout [HKO], and complement [CM] system for GAPDH). By performing various *in vitro* and *in vivo* experiments with these cell lines, we are trying to bridge between two phenomena: EVs–mediated LmGAPDH secretion during *Leishmania* infection and the regulation of the first line of host defense.

## Results

### Localization of LmGAPDH in *Leishmania* promastigotes

It is well established that the cytosolic human GAPDH enzymes are targeted to the plasma membrane (21, 32) and the nucleus (33) for showing moonlighting activity. Although both LmGAPDH copies contain the predicted C-terminal glycosomal tri-peptide (PKL) signal sequence, the enzyme may be present in other organelles for displaying nonglycolytic functions. To ascertain whether the mature LmGAPDH protein was localized in any specific organelles other than the glycosome as a native protein, homogenates of *L. major* cells were fractionated by differential centrifugation, glycosomes were isolated from *L. major* promastigotes using Colasante *et al.*'s (34) technique, and *Leishmania*-secreted microvesicles were harvested following the conventional EVs isolation protocol from conditioned medium subjecting to ultracentrifugation with prior incubation of the promastigotes at 37 °C for ∼2 h.

**Figure 1:**
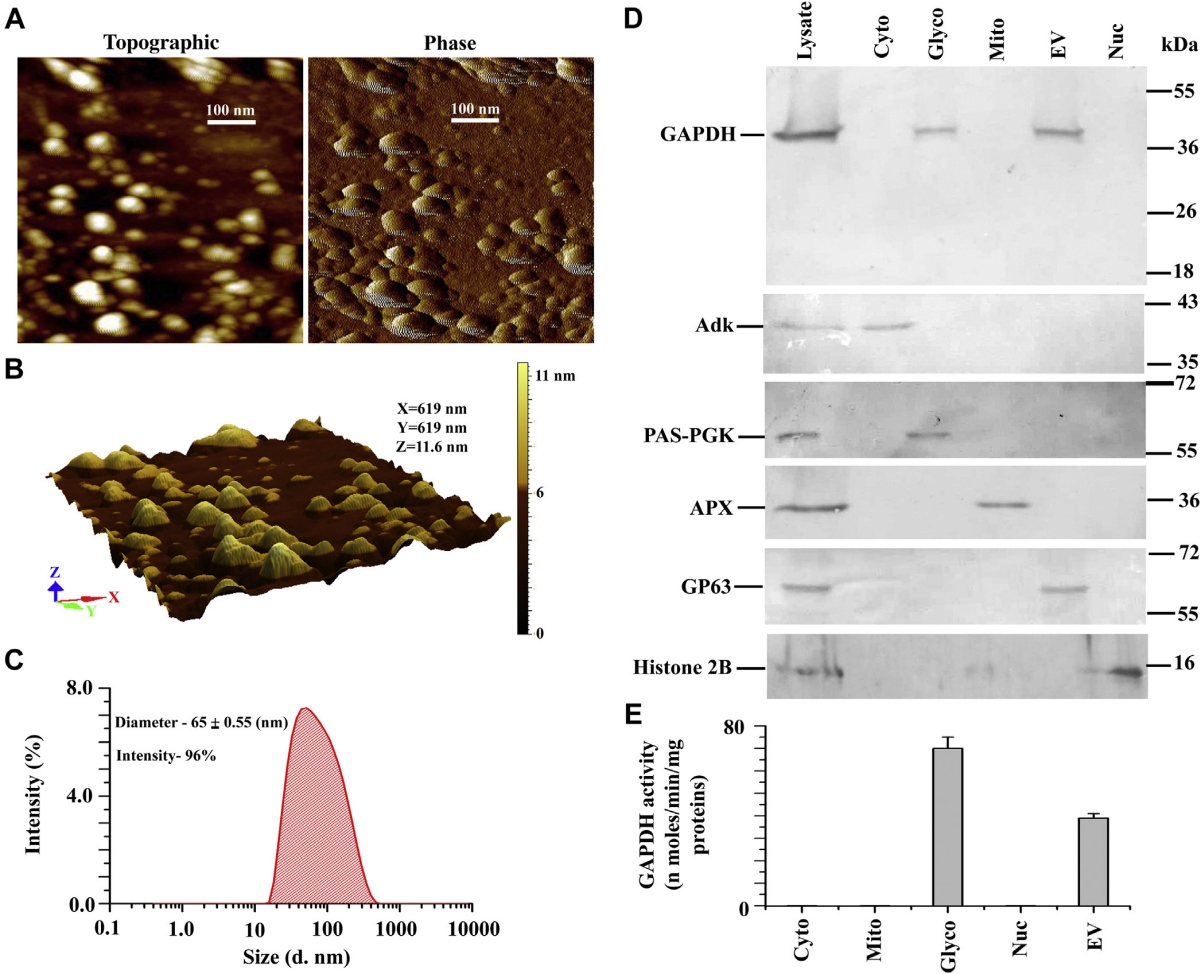
**Localization of LmGAPDH.***A*, the shape and size of extracellular vesicles (EVs) extracted from *L. major* were identified through atomic force microscopy (topography and phase mode). *B*, three-D image of same EVs as in *A*. *C*, the average diameter of *Leishmania* EVs was determined by dynamic light scattering. *D*, subcellular localization of LmGAPDH was performed by Western blotting. Lanes: 1, cell lysate; 2, cytosolic; 3, glycosomal; 4, mitochondrial; 5, EVs; and 6, nuclear fraction. Adenosine kinase, PAS domain containing phosphoglycerate kinase, ascorbate peroxidase, GP63, and histone 2B were used in the subcellular fractions as cytosolic, glycosomal, mitochondrial, EVs and nuclear markers, respectively. *E*, the enzymatic activity of LmGAPDH in different subcellular fractions. Cyto, glyco, mito, ev, and nue denote as cytosolic, glycosomal, mitochondrial, EVs, and nuclear fraction, respectively.

Then, we verified the morphology and size of the EVs *via* atomic force microscopy (AFM) (Fig. 1, *A* and *B*). Two-dimensional (tapping mode topographic and phase AFM) and three-dimensional views confirmed the spherical morphology and size of the vesicles to around 65 ± 5 nm in diameter, which were identical to EVs' previously recognized form (35). To further confirm these data, dynamic light scattering (DLS) analysis was performed. The results obtained from DLS confirmed that the mean diameter of the EVs derived from *Leishmania* was ∼65 nm with high homogeneity (96%) (Fig. 1*C*). Western blot results showed that the LmGAPDH protein band was recovered from both glycosomal and EVs fractions, where the glycosomal PAS domain containing phosphoglycerate kinase and the EVs GP63 protein (as a marker protein) were concentrated (Fig. 1*D*), indicating that the enzyme is localized in both the glycosome and the EV of *Leishmania*. On the other hand, the LmGAPDH protein band was absent in the cytosol, mitochondria, and nucleus fractions, where the cytosolic adenosine kinase, mitochondrial ascorbate peroxidase, and nuclear histone H2B (as a marker protein) were concentrated. Similarly, the GAPDH activity in the crude extract was recovered from both the glycosomal and the EVs fraction, suggesting that the enzyme is almost entirely localized in the glycosome and the EV of *Leishmania* (Fig. 1*E*).

### Analysis of EV mediate LmGAPDH in overexpressed, control, half knockout, and complement cells

**Figure 2:**
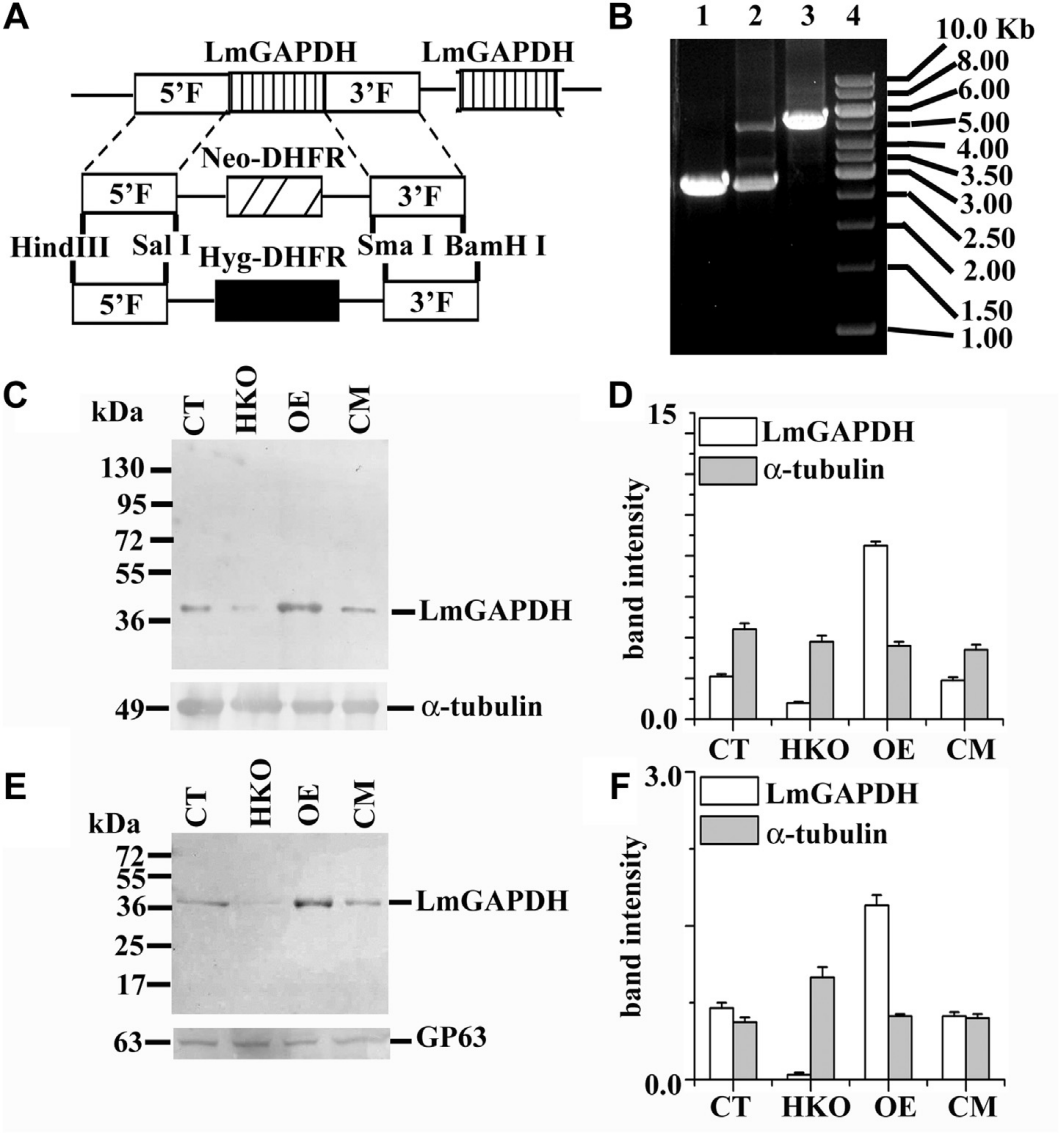
**Targeted gene replacement of single copy of LmGAPDH gene.***A*, schematic representation of the LmGAPDH locus and the plasmid constructs used for gene replacement. DHFR denotes dihydrofolate reductase. *B*, agarose gel analysis of PCR-amplified products of LmGAPDH gene. Lane 4 shows the molecular mass marker, lanes 1, 2, and 3 correspond to PCR with genomic DNA from CT, +/− allele, and HKO mutants, respectively, with external (5′ and 3′ flanking region) primers to the LmGAPDH gene. The expected sizes of the LmGAPDH, NEO, and HYG gene PCR product are 2.75, 5.3, and 5.7 kb, respectively. *C*, *L. major* lysate was used for Western blotting. Western blot results using rabbit anti-LmGAPDH and mouse anti-tubulin antibody are shown. *D*, bar diagram depicted as the percentage of band intensities in *C*. *E*, *L. major* extracellular vesicles were used for Western blotting. Western blot results using rabbit anti-LmGAPDH and mouse anti-GP63 antibody are shown. *F*, bar diagram denoted as the percentage of band intensities in *E*. Band intensity was quantified by ImageJ software (NIH). OE, CT, CM, and HKO denote overexpressed, control, complement, and half knockout cell lines.

*L. major* Gene DB reveals that the LmGAPDH gene has its two identical copies in tandem array. To investigate the nonglycolytic role of LmGAPDH gene in *L. major*, a gene replacement technique was carried out. Since the LmGAPDH is an essential gene for the survival of *Leishmania* parasite, two copies of the gene cannot be deleted. Thus, we knocked out only one of the two copies of the gene, denoted as HKO. We generated LmGAPDH HKO constructs by gene replacement of hygromycin and neomycin selectable markers (Fig. 2*A*). After two rounds of transfection and selection with neomycin and hygromycin drugs, both the alleles of a single copy of LmGAPDH gene in cells had been replaced. To screen the HKO mutants, we first performed a PCR analysis on genomic DNA with primers generated from the 5′- and 3′-flanking regions (Fig. 2*B*); then Western blot analysis with anti-LmGAPDH antibody further confirmed the presence of ∼50% LmGAPDH in the HKO cell line (Fig. 2, *C* and *D*). These results suggested that both alleles of a single copy of LmGAPDH gene had been knocked out in the *L. major* cell line that is resistant to both neomycin and hygromycin drugs. On the other hand, the results of Western blot technique demonstrated higher level of LmGAPDH protein expression (4.5-fold) in OE cells compared with CT, even though the amount of LmGAPDH in CM and CT is equivalent (Fig. 2, *C* and *D*). Next, we investigated whether the level of LmGAPDH was changed in the EVs isolated from HKO and OE cells compared with CT and CM. Western blotting confirmed that the EVs from HKO cells had negligible amount of LmGAPDH protein (Fig. 2, *E* and *F*), whereas the EVs from the OE cell line had expressed an ∼2.3 times higher amount of LmGAPDH compared with CT or CM.

### Characteristics of OE, CT, HKO, and CM cells

**Figure 3:**
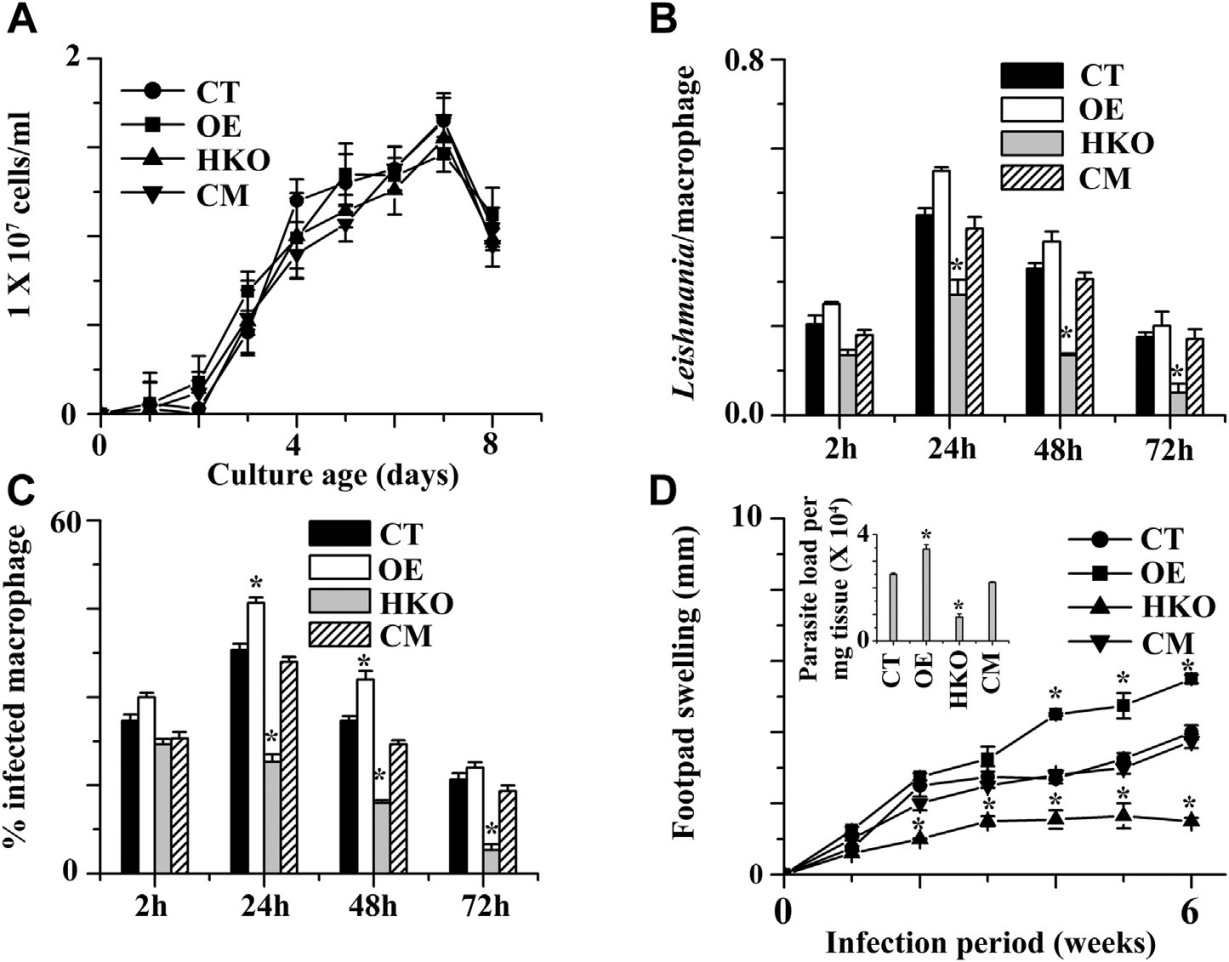
**Functional characterization of LmGAPDH variant *L. major* cells.***A*, the growth curves of CT, OE, CM, and HKO cells. *B*, the percentage of macrophages infected with CT, OE, CM, and HKO parasites. For each time point, 200 macrophages were counted. *C*, the number of *Leishmania* within each infected macrophage was counted. For each time point and cell type, 200 infected macrophages were analyzed. *D*, infection in BALB/c mice. Footpad swelling of CT, OE, CM, and HKO was observed for the three groups (15 mice/group). *D*, *inset*, parasite burden in the footpad after 6 weeks post infection in CT, OE, CM, and HKO cells. ∗Statistically significant value of less than 0.05. OE, CT, CM, and HKO denote overexpressed, control, complement, and half knockout cell lines.

To investigate whether the growth rate of HKO mutants is similar to CT, CM, or OE promastigotes, microscopically viable cell counting analysis was performed. The growth curve showed that the HKO population had no significant change in the growth rate compared with CT, CM, or OE cells (Fig. 3*A*). Thus, a single copy of GAPDH in HKO expresses sufficient amounts of proteins that maintain glycolytic function of promastigotes for growth. Because *L. major* promastigotes can infect host macrophages, we investigated the interaction of promastigotes with the macrophages. We explored to what extent HKO, CT, CM, and OE cells were endocytosed by the macrophages. The adhering rates of all promastigotes were more or less same after 2 h of incubation period (Fig. 3*B*). HKO promastigotes, on the other hand, internalized at a substantially lower rate than WT, OE, or CM cells after 24 and 48 h of incubation. Most infected macrophages still had parasites 72 h after phagocytosing WT and CM promastigotes, whereas the percentage of parasites in HKO infected macrophages dropped considerably. These results indicate that the HKO parasites were killed more easily inside the macrophages. In addition, the percentage of macrophages infected with OE increased considerably (Fig. 3*C*) compared with CT infection. Similarly, mice infection results suggested that HKO cells could not develop a severe disease, with an earlier onset of footpad necrosis, compared with CT or CM promastigotes (Fig. 3*D*). These results were supported by the OE promastigotes, showing increased virulence in *in vivo* mice model. The result of parasite burden during 6 weeks post infection indicated that HKO parasites, compared with CT or CM, had about 2.6-fold less parasite burden (Fig. 3*D* inset) in 1 mg of footpad tissue. These findings indicated that the GAPDH gene in parasites plays an important role in macrophage infection and disease development in mice.

### Deletion of single copy of LmGAPDH gene leads to changed expression of TNF-α in host macrophage

**Figure 4:**
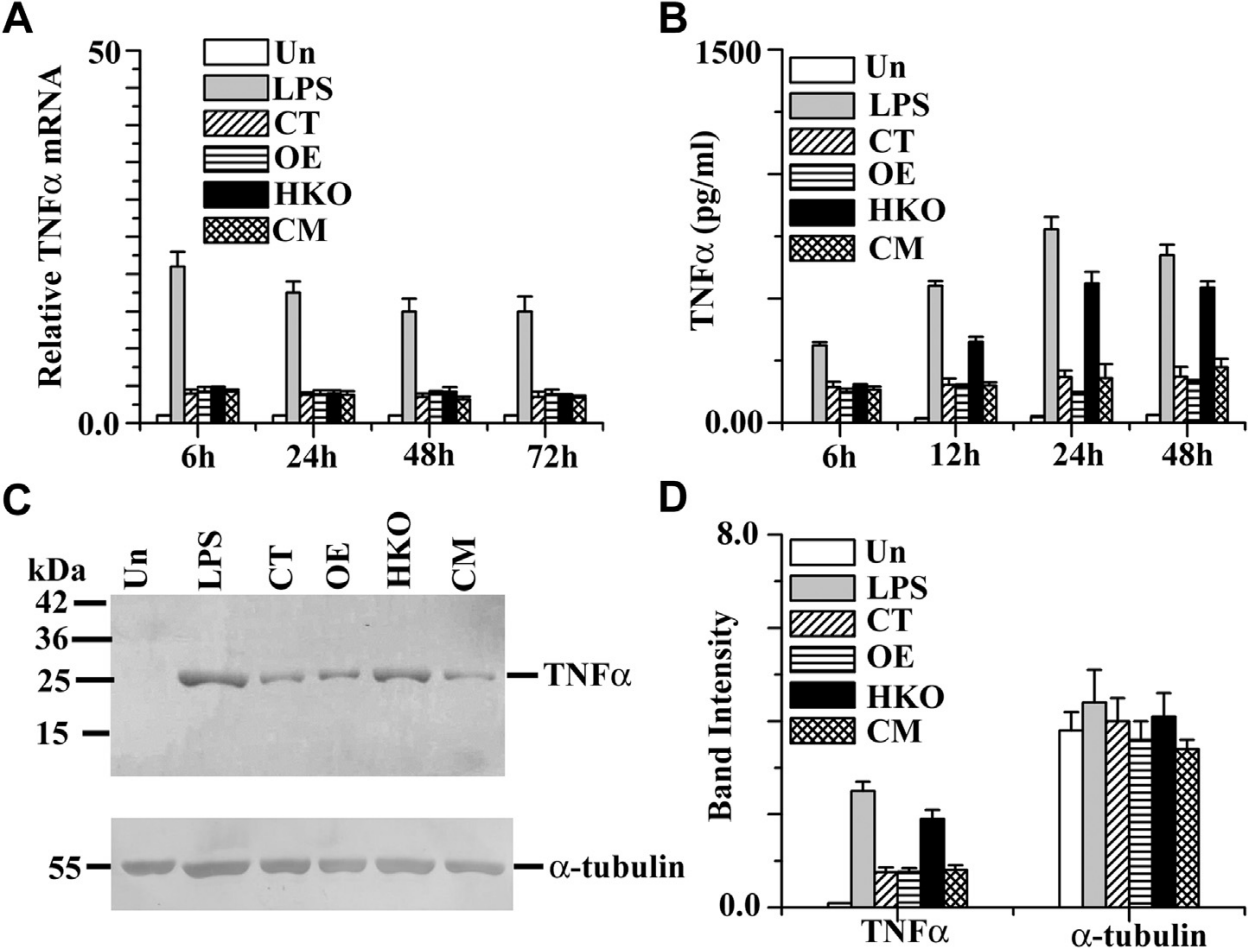
**mRNA and protein expression of TNF-α production in stationary-phase *Leishmania* promastigote-infected macrophage.***A*, measurement of gene transcript abundance was analyzed by using quantitative real-time PCR as described in Experimental procedures. All data were normalized using beta-actin as the endogenous control. *B*, levels of TNF-α production were measured by ELISA in the cell culture supernatants after infection. *C*, TNF-α protein levels were measured by Western blot analysis of protein lysates from RAW264.7 cells. Alpha tubulin was used as loading control. *D*, densitometric analysis of all bands was done using ImageJ software (from NIH). All data are representative of three independent experiments. Error bars represent the SD from three independent experiments. “Un” denotes uninfected macrophage (-*Leishmania*). LPS denotes induced macrophage with LPS (1 μg/ml). OE, CT, CM, and HKO denote overexpressed, control, complement, and half knockout cell lines.

TNF-α, a proinflammatory cytokine, appears to have a crucial role in the regulation of infection. GAPDH has been found to influence inflammatory TNF-α and immunological responses by modulating macrophage activity (36). TNF-α expression in OE, CT, CM, and HKO infected macrophages was determined by quantitative real-time PCR assay (Fig. 4*A*), ELISA (Fig. 4*B*), and Western blot analysis (Fig. 4, *C* and *D*). Quantitative real-time PCR assay showed that the TNF-α mRNA expression in the HKO infected macrophage was unaltered compared with CT, OE, and CM infected cells (Fig. 4*A*) up to 72 h infection period. Conversely, ELISA data suggested that the HKO infected macrophage cells produced a higher amount of TNF-α (∼3-fold) expression (Fig. 4*B*) compared with CT and CM cells. As expected, lipopolysaccharide (LPS) (1 μg/ml) induced both mRNA and protein level of TNF-α expression in macrophages. To further confirm these data, Western blot analysis was performed (Fig. 4, *C* and *D*), and the results we got were almost similar to the ELISA data. Altogether, these results suggest that the LmGAPDH regulates the host TNF-α expression at the translation level.

**Figure 5:**
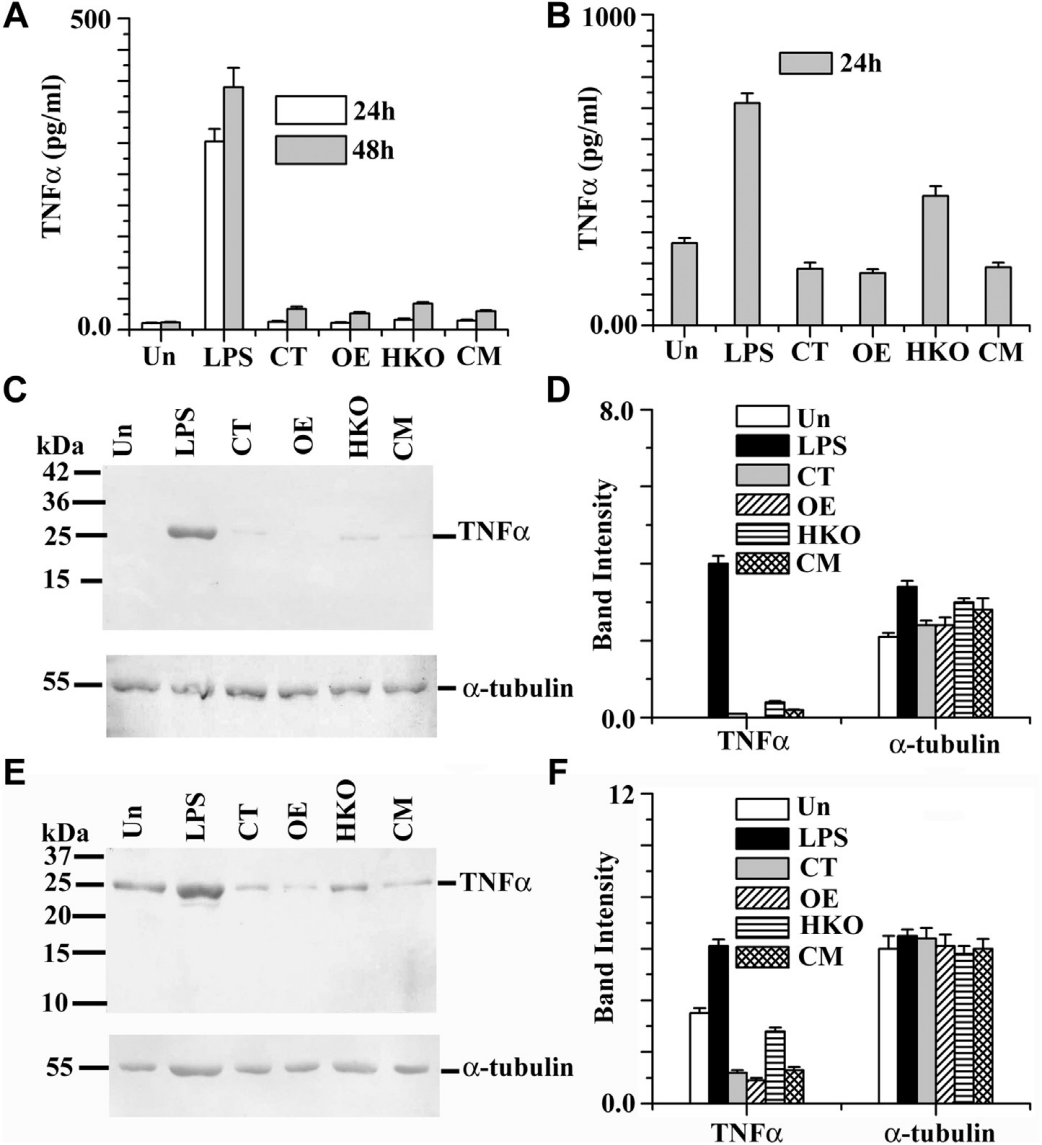
**TNF-α expression in the extracellular vesicles–treated uninduced and induced macrophages.***A*, levels of TNF-α secretion were measured by ELISA in the cell culture supernatants of extracellular vesicles–treated uninduced macrophage. *B*, levels of TNF-α secretion were measured by ELISA in the cell culture supernatants of extracellular vesicles-treated 0.1 μg/ml LPS preinduced macrophage. *C*, TNF-α protein levels were measured by Western blot analysis of protein lysates from extracellular vesicles–treated uninduced RAW264.7 cells. Alpha tubulin was used as loading control. *D*, densitometric analysis of all bands of C was done using ImageJ software (from NIH). *E*, TNF-α protein levels were measured by Western blot analysis in extracellular vesicles–treated 0.1 μg/ml LPS preinduced RAW264.7 cells. Alpha tubulin was used as loading control. *F*, densitometric analysis of all bands of *E* was done using ImageJ software (from NIH). All data are representative of three independent experiments. Error bars represent the SD from three independent experiments. “Un” denotes macrophage lysate without extracellular vesicles treatment. LPS denotes LPS-induced macrophage (1 μg/ml). OE, CT, CM, and HKO denote overexpressed, control, complement, and half knockout cell lines.

To check the effect of the LmGAPDH-deficient EVs in the host TNF-α expression, we measured the TNF-α expression in EVs (from OE, CT, CM, or HKO) treated macrophages by ELISA (Fig. 5, *A* and *B*) and Western blot (Fig. 5, *C*–*F*). ELISA data suggested that the EVs from HKO treated uninduced macrophage showed very little change in TNF-α expression compared with the EVs from CT or CM treated cells (Fig. 5*A*) up to 48 h incubation periods. In case of 0.1 μg/ml LPS preinduced macrophage, the EVs from OE, CT, or CM cells suppressed the TNF-α expression, whereas the EVs from HKO cells fail to represses TNF-α expression (Fig. 5*B*). To further confirm these data, Western blot analysis was performed (Fig. 5, *C*–*F*), and the outcomes were nearly identical to the ELISA data. Altogether, these results suggest that the GAPDH in parasite's EVs may regulate TNF-α expression when they interacted with the host macrophage.

**Figure 6:**
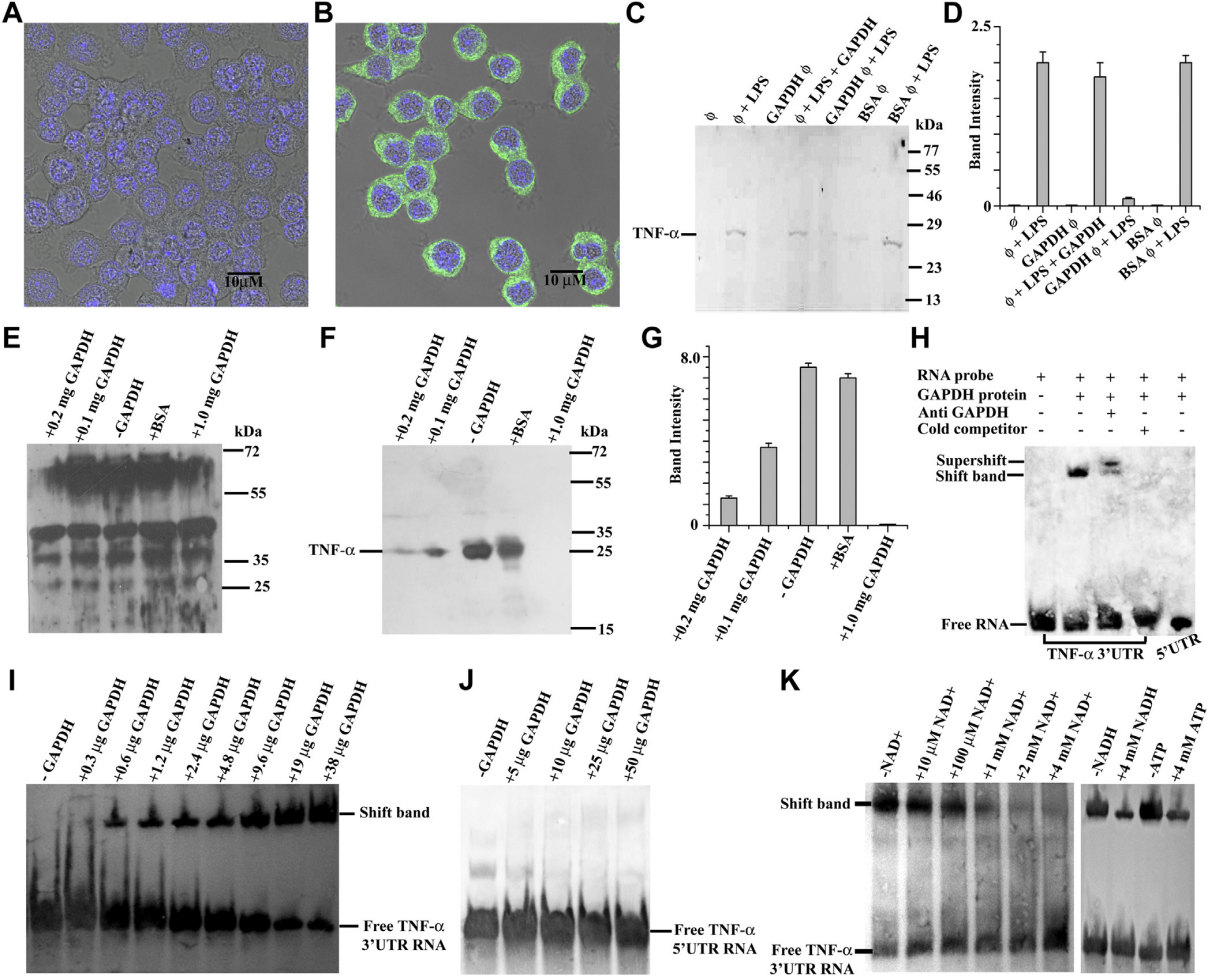
**Quantitation of TNF-α repression by purified LmGAPDH and its binding to the 3′ UTR region of TNF-α mRNA.** Visualization of normal macrophage (*A*) and LmGAPDH-transfected macrophage (*B*) by confocal microscope in bright field background. Nucleus was stained with DAPI (*blue*). Anti-rabbit LmGAPDH antibody and Alexa Fluor 488–conjugated anti-rabbit secondary antibody (Thermo Scientific) were used for visualization of LmGAPDH. *C*, the levels of TNF-α protein expression were measured from LmGAPDH-transfected RAW264.7 cells in presence of LPS. Φ, GAPDH ϕ, and BSA ϕ denote macrophage, LmGAPDH-transfected macrophage, and BSA-transfected macrophage cells. *D*, densitometric analysis of all bands of C was done using ImageJ software (from NIH). *E*, the autoradiographic image of *in vitro* general translation pattern. *F*, the autoradiographic image of *in vitro* TNF-α translation with respect to various concentrations of LmGAPDH. *G*, densitometric analysis of all bands of *F* was done using ImageJ software (from NIH). *H*, the REMSA measurement for LmGAPDH binding to the 3′UTR region of TNF-α mRNA. *I*, the RNA electrophoretic mobility shift assay image for LmGAPDH–3′UTR TNF-α mRNA complexes with respect to various concentrations of LmGAPDH. *J*, the RNA electrophoretic mobility shift assay image for the binding pattern of 5′UTR TNF-α mRNA with various concentrations of LmGAPDH. *K*, inhibition of 3′UTR TNF-α mRNA binding of LmGAPDH by NAD^+^, NADH, and ATP.

It is well defined that EV has a lot of components including LmGAPDH; thus, we checked whether recombinant LmGAPDH itself could block the host TNF-α expression. To ensure recombinant LmGAPDH transfection within the macrophage, the DAPI-stained LmGAPDH-transfected macrophages were costained with rabbit anti-LmGAPDH as primary antibody and Alexa Fluor 488–conjugated anti-rabbit secondary antibody (Fig. 6, *A* and *B*). Figure 6*B* confirmed the presence of recombinant LmGAPDH in the cytosol. Western blot analysis confirmed that LmGAPDH-transfected RAW 264.7 cells showed lower level of TNF-α expression in the presence of LPS (Fig. 6, *C* and *D*) compared with bovine serum albumin (BSA)-transfected RAW 264.7 cells. These results directly proved that LmGAPDH is the actual factor for the suppression of TNF-α expression. Then, we investigated *in vitro* protein translation of TNF-α in the presence or absence of purified LmGAPDH (Fig. 6, *E* and *F*). The gel autoradiography analysis showed that the protein synthesis band pattern in all lanes is similar (Fig. 6*E*), indicating that *in vitro* protein translation occurred in the presence or absence of purified LmGAPDH. After immunoprecipitation with TNF-α monoclonal antibody, the gel showed the TNF-α expression is inversely proportional to the LmGAPDH concentration (Fig. 6, *F* and *G*). In contrast to LmGAPDH, BSA protein could not suppress *in vitro* TNF-α protein translation. GAPDH also acts as a specific mRNA-binding protein that interacts with 5′ or 3′-UTR regions of mRNA regulating gene expression positively or negatively at the posttranscriptional level (14). Our data already indicate that LmGAPDH is responsible for the decrease in TNF-α protein expression. To further demonstrate that this decrease in cytokine production is due to posttranscriptional repression, we examined the binding of 5′-UTR or 3′-UTR of TNF-α mRNA by LmGAPDH (Fig. 6*H*) using an RNA electrophoretic mobility shift assay (REMSA). A labeled oligonucleotide corresponding to the 3′-UTR of TNF-α mRNA produced a retarded band with LmGAPDH that may be an inactive form of mRNA–LmGAPDH complexes (Fig. 6*H*). This retarded band could be competed with a 200-fold molar excess of the unlabeled homologous oligonucleotide. When anti-LmGAPDH antibody was added to the REMSA mixture, the 3′-UTR of TNF-α mRNA–LmGAPDH–anti-LmGAPDH antibody complex moved more slowly down the REMSA gel, indicating that the complex was more likely to be super shifted. In comparison with 3′-UTR of TNF-α mRNA, the shifted band was not observed in case of 5′-UTR of TNF-α mRNA oligonucleotide. Figure 6*I* showed that the binding of 3′-UTR of TNF-α mRNA with LmGAPDH is concentration dependent. Conversely, 5′-UTR of TNF-α mRNA could not form complex with LmGAPDH at higher concentrations also (Fig. 6*J*). These findings suggest that LmGAPDH binds with the 3′-UTR of TNF-α mRNA, preventing the transcript from being translated and hence decreasing TNF-α cytokine production. To identify the interaction site of LmGAPDH with 3′-UTR of TNF-α mRNA, we examined the competitive assay with cofactor NAD^+^/NADH or ATP in 3′-UTR–LmGAPDH complexes (Fig. 6*K*) by RNA electrophoretic mobility shift assay. Increasing concentrations of NAD^+^, NADH, and ATP decreased GAPDH binding ability toward 3′-UTR. These results indicate that cofactor NAD^+^-interacting site of LmGAPDH is responsible for 3′-UTR binding.

## Discussion

Recently several studies demonstrated that GAPDH is translocated to different subcellular organelles for exhibiting nonglycolytic function (18). Hence a precise knowledge about the localization of LmGAPDH will aid in predicting the potential role of this protein and enabling us to correlate its functions with those of other proteins that may be engaged in a different cellular signaling pathway for parasite survival. It is well known that the glycosomal and the cytosolic GAPDH play a major role in glycolytic function in trypanosomatid. Here we have shown for the first time by using various molecular approaches (including Western blot analysis, and activity measurement of protein) that the localization of LmGAPDH is in the glycosomal fraction as well as in the EV fraction, similar to where the leishmanial GP63 protein is found (37). Hence, EV mediate LmGAPDH is likely to affect the host defense system directly. The existence of an EV mediate LmGAPDH raises the question how this protein is translocated to EVs. LmGAPDH lacks an N-terminal secretion signal peptide and is thus thought to be secreted *via* nonconventional mechanisms. A similar type of result was found in proteomic study on *Leishmania*, where majority of the temperature shift–induced secreted proteins lacked the N-terminal signal peptide (36).

During the past two decades, several secretory virulent proteins have been identified in *Leishmania* EVs, including GP63 (37, 38), heat shock protein 100 (39), proteophosphoglycan pPPG2 (40), acid phosphatase (41), heat shock protein 70 (42), EF1alpha (43), and fructose-1,6-bisphosphate aldolase (44). In addition, a group of workers have demonstrated that leishmanial EVs from the extracellular environment is taken up by naive host cells, which selectively induced IL8 secretion (1). These data suggest that *Leishmania* use EVs to deliver effector molecules to host cells as well as to communicate with the host cellular environment. In this study, we describe how *Leishmania* parasite influences host TNF-α protein expression through a mechanism not previously observed in the macrophage. Here, the main finding is that the EVs-derived LmGAPDH from *Leishmania* promastigotes selectively downregulate host TNF-α production suppressing the host immune system. Furthermore, our data on *Leishmania*-infected macrophages and mouse models also indicate that LmGAPDH is essential for the survival of *L. major* parasites within macrophage and virulence in mice.

EVs released from promastigotes have freedom to interact with host cells, by receptor binding, fusion with the plasma membrane, or through endocytosis. So LmGAPDH may be active in host cells on the surface of the host plasma membrane, in the cytosol, or within the host phagolysosome. LmGAPDH-transfected macrophage showed a lower level of TNF-α expression in the presence of LPS (lane 5 in Fig. 6*C*) compared with BSA-transfected RAW 264.7 cells (lane 7 in Fig. 6*C*), but naked LmGAPDH cannot suppress TNF-α expression in LPS-treated macrophages (lane 4 in Fig. 6*C*). These results suggest that LmGAPDH has to penetrate the host membrane for blocking TNF-α expression. The general concept is that the virulence factors secreted by most of intracellular pathogens target the host's cytosolic molecules for remodeling host cell function (45, 46). Now one question that immediately arises is which step is apparently involved in TNF-α suppression. First, *Leishmania*-derived GAPDH may either interact with a host transcription factor or bind to the promoter region of TNF-α gene and as a result suppress host TNF-α at the level of transcription. The second possibility is that the regulation of TNF-α expression might be affected at the posttranscriptional level. Like LPS, RT-PCR results suggest that *Leishmania*-infected macrophage produces a higher amount of TNF-α mRNA compared with uninfected cells. The RT-PCR results from the comparative studies among various concentrations of GAPDH containing *Leishmania* (OE, CT, HKO, CM)-infected macrophages suggest that the transcription of TNF-α mRNA from host DNA did not depend on LmGAPDH concentration. So we can rule out the first possibility. On the other hand, ELISA assay and immunoblot data suggest that the protein expression of TNF-α in infected host cells is inversely proportional to the LmGAPDH concentration. Despite showing no difference in TNF-α mRNA, however, infected host cells show a significant change in TNF-α protein expression. These results immediately demonstrated the relevance of posttranscriptional repression of TNF-α mRNA. Recently, it was shown that GAPDH could bind with AU-rich elements of the untranslated region of mRNA that is responsible for posttranscriptional regulation of IFN-γ (47) and TNF-α expression, inhibiting translation and limiting IFN-γ and TNF-α cytokine production (48, 49, 50). Our mRNA (AU-rich elements of untranslated region)-binding assay suggests that LmGAPDH can bind with AU-rich elements of 3′ UTR of TNF-α.

This raises the question as to which region of LmGAPDH is implicated in binding to AU-rich elements of 3′ UTR of TNF-α. It is known that incubation of GAPDH with NAD+ decreases its RNA-binding activity (14). Our competition studies suggest that NAD+, NADH, and ATP were able to diminish the specific 3′ UTR binding of LmGAPDH indicating that the dinucleotide-binding region in the Rossmann fold of LmGAPDH might serve as a 3′ UTR–binding domain. Our data predict that the Rossmann fold of LmGAPDH might be reciprocally regulated between its 3′ UTR binding (inactive in glycolysis) and NAD+ binding (active in glycolysis) states within the macrophage. This mechanism would favor glycolytic activity of LmGAPDH in the parasite glycosome (where the concentration of NAD+ is high). Conversely, LmGAPDH may bind host TNF-α mRNA during *Leishmania* infection due to metabolic reprogramming of *Leishmania*-infected human macrophage, which results in increased oxidative phosphorylation relative to glycolysis and to lower concentrations of NAD+/NADH (51).

How do the LmGAPDH–TNF-α mRNA complexes inhibit translation of the transcript and limiting TNF-α production? *In vitro* protein translation results suggest that the protein expression of TNF-α decreases with increasing LmGAPDH concentration. On the other hand, BSA fails to repress *in vitro* TNF-α protein translation. These results confirm that the LmGAPDH–TNF-α mRNA complexes inhibit translation of TNF-α expression.

**Figure 7:**
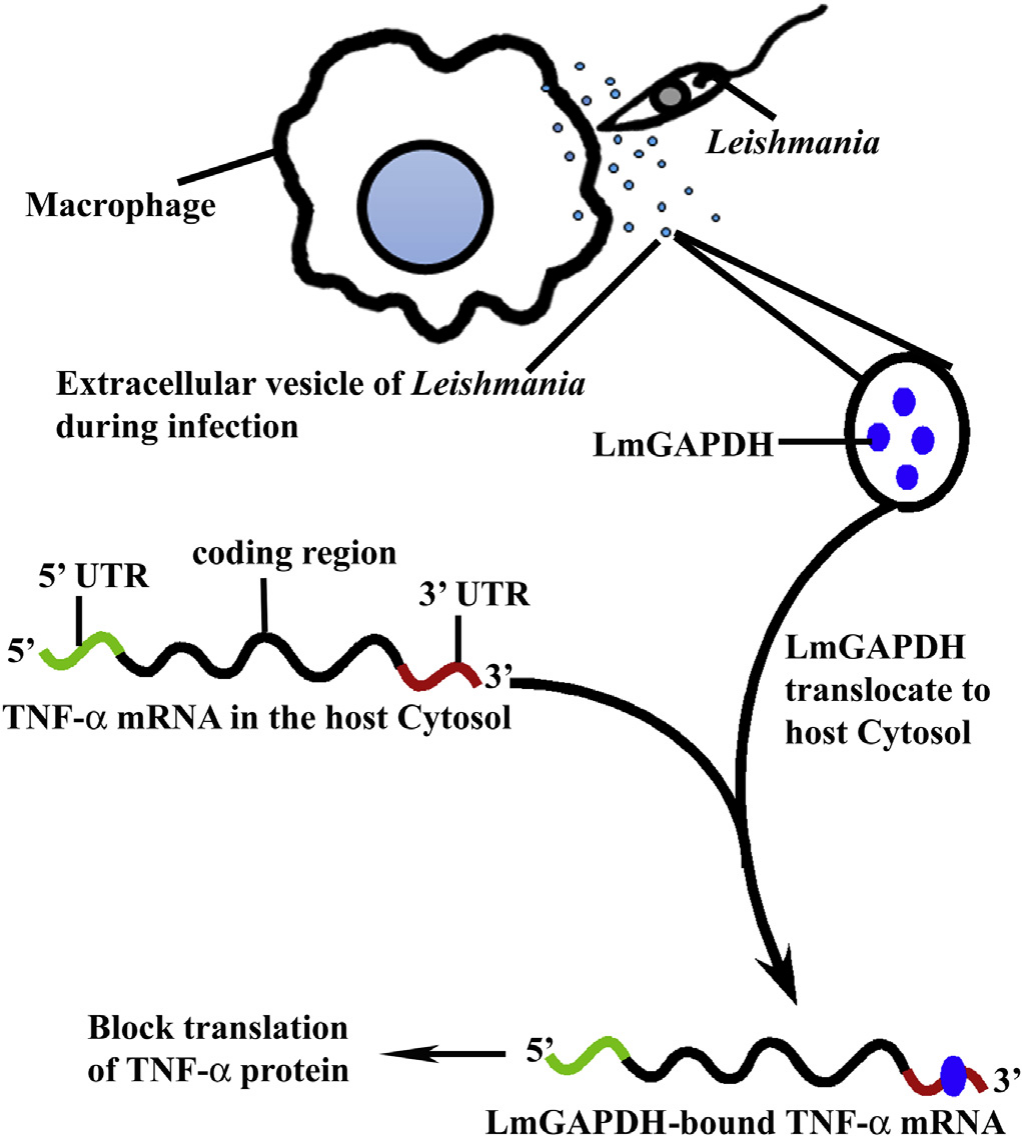
**A possible schematic diagram for a novel virulence function of LmGAPDH contained in extracellular vesicle**.

In order to explain the possible mechanism for the suppression of TNF-α in *Leishmania*-infected macrophage cells, we propose a mechanism (shown schematically in Fig. 7) based on a novel virulence function of LmGAPDH contained in EV that is translocated from parasite to host macrophage during infection. Our results show for the first time that the glycolytic enzyme LmGAPDH posttranscriptionally regulates macrophage proinflammatory function by binding to the AU-rich region in the 3′ UTR of TNF-α mRNA and reducing protein translation and thereby modulates host immune response. Thus, targeting LmGAPDH gene present in EV could be a potential arena for medication development in the near future in the fight against leishmaniasis.

## Experimental procedures

### Reagents

*L. major* (strain 5ASKH) was procured from the *Leishmania* strain bank of our Institute. All reagents and materials were purchased from Sigma or sources reported previously (52, 53, 54).

### Ethics statement

All BALB/c mice and rabbits were obtained from and maintained in our institutional animal facility (Kolkata, India). The research project was approved by the IICB Animal Ethical Committee (Registration No.147/1999, CPCSEA) and registered with the Committee for the Purpose of Control and Supervision on Experiments on Animals (CPCSEA), Government of India. The BALB/c mice and rabbits were handled according to their guidelines.

### Parasite culture

*L. major* wildtype parasites were routinely cultured at 22 °C in M199 medium (Invitrogen) supplemented with 40 mM Hepes (pH 7.4, Amresco), 200 μM adenine (Sigma), 1% penicillin–streptomycin (v/v), 50 μg/ml gentamycin (Abott), and 10% heat-inactivated fetal bovine serum (Invitrogen).

### Macrophage culture

The murine macrophage cell line RAW 264.7 maintained in RPMI-1640 medium (Gibco) containing 10% heat-inactivated fetal bovine serum at 37 °C in a humidified atmosphere containing 5% CO_2_ was routinely passaged using a cell scraper and replated in tissue culture flasks at a ratio of 1:6 when they reached ∼80% confluence.

### Genomic DNA isolation from *L. major*

Genomic DNA was isolated from *L. major* logarithmic phase promastigotes by using Qiagen genomic DNA isolation kit at room temperature (25 °C). The concentration was 25 ng/μl.

### Cloning of LmGAPDH

Using the forward primer 5′- AAAACATATGATGGCTCCTATCAAGGTCGG-3′ and reverse primer 5′-AAAAGGATCCCTACATCTTGGCGCTCGCAG-3′, the entire ORF of the GAPDH (accession no: Lmjf.30.2970) was amplified to get a 1083-bp fragment that was digested and cloned within the pET15B vector in NdeI/BamHI restriction sites. DNA sequencing was performed to confirm the ORF.

### Expression and purification of LmGAPDH

**Figure 8:**
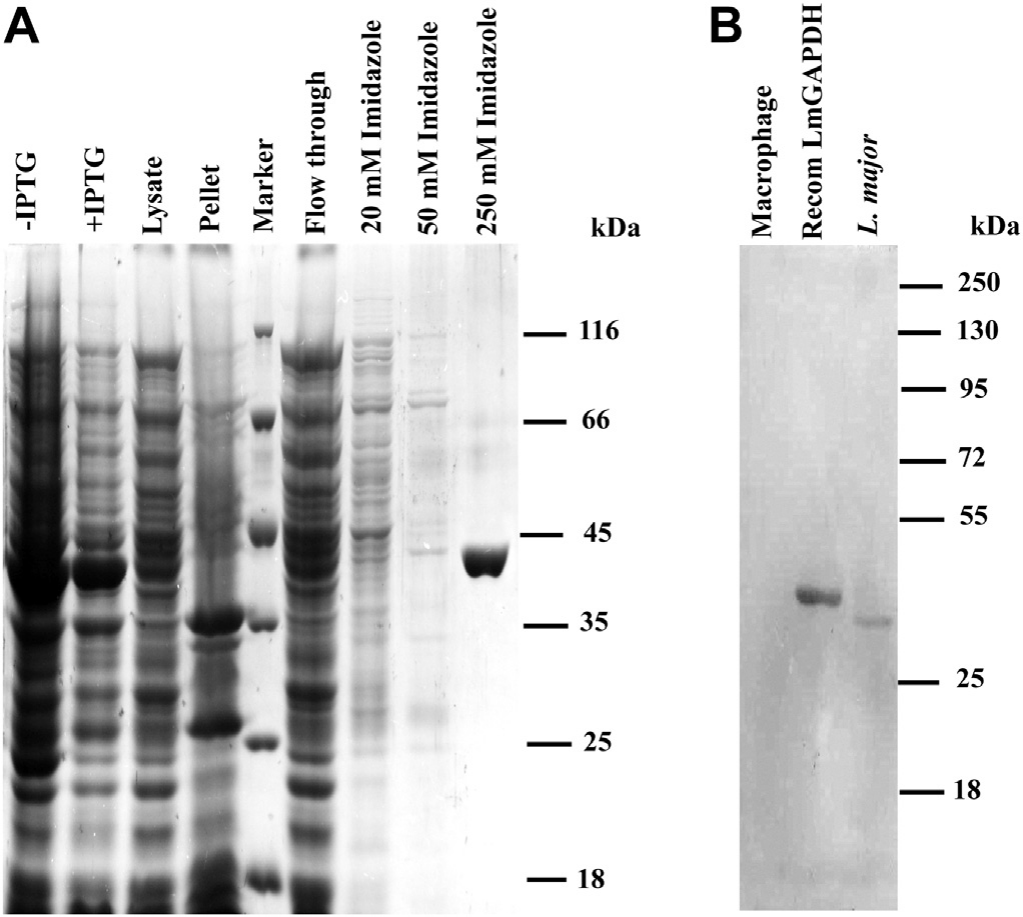
**Measurement of specificity of polyclonal anti-LmGAPDH antibodies against LmGAPDH.***A*, SDS–PAGE during recombinant LmGAPDH purification. *B*, Western blot for macrophage cell lysate and *L. major* cell lysate by using polyclonal anti-LmGAPDH antibodies.

Recombinant pET15B/LmGAPDH were transformed into *Escherichia coli* BL21(DE3) and were grown overnight in 100 ml of Luria–Bertani broth containing 100 μg/ml ampicillin at 37 °C shaking. The overnight grown culture was then inoculated in 1 L of terrific broth. When the culture reached an absorbance of around 0.8 to 1.0 at 600 nm, 0.25 mM isopropyl β-D-1-thiogalactopyranoside was added and bacteria were further grown at 28 °C for around 18 h. Cells were then harvested by centrifugation at 6000*g* for 10 min and washed two times with 1× PBS. Purification of the recombinant LmGAPDH was achieved by a chromatographic step, which used nickel–nitrilotriacetic acid–agarose. The protein compositions of samples obtained from the various purification steps are shown in Figure 8*A*. As expected, purification yielded a single protein band of approximately 39 kDa. No other protein band could be detected in the gel stained with Coomassie brilliant blue.

### Production of polyclonal antibodies against LmGAPDH

Polyclonal antibodies against the purified recombinant LmGAPDH (20 μg) were raised by subcutaneous injection in 6-month-old female rabbit using Freund's complete adjuvant. This was followed by three booster doses of recombinant LmGAPDH (15 μg) with incomplete adjuvant at 2-week intervals. The rabbit were bled 2 weeks after the last booster, and sera were collected and used for Western blot analysis (Fig. 8*B*).

### Enzyme assay using UV–visible spectroscopy

LmGAPDH activity was measured spectrophotometrically at 25 °C using Shimadzu UV-2550 spectrophotometer by monitoring NADH generation at 340 nm wavelength. The assay mixture was composed of 40 mM Tris-HCl buffer (pH 7.5), 50 mM K_2_PO_4_, 0.15 mM NAD^+^, and 2.5 mM glyceraldehyde 3-phosphate. After incubating in a 1.0-ml cuvette at 25 °C for 5 min to achieve temperature equilibrium and establish blank values, the reaction was initiated with the addition of enzyme. Absorbance from 0 to 3 min was recorded.

### Construction of overexpression system for LmGAPDH in *Leishmania*

Primers 5′-AAAACCCGGGATGGCTCCTATCAAGGTCGG-3′ (forward) and 5′-AAAAGGATCCCTACATCTTGGCGCTCGCAG-3′ (reverse) were used to amplify the ORF of *L. major* GAPDH gene by PCR. The amplified portion was then cloned into the SmaI/BamHI site of pXG-B2863 vector, which was transfected in *L. major* by electroporation (55). Overexpressed cells were maintained at 200 μg/ml neomycin.

### Preparation of control cell lines

Only pXG-B2863 vector was transfected in *L. major* by electroporation. Control cells were maintained at 200 μg/ml neomycin.

### Generation of half knockout strain for LmGAPDH alleles

Modified pXG-neo and pXG-hyg vectors were used to generate the HKO constructs of LmGAPDH gene. Primers 5′-AAAAAAGCTTAGCTGACCGGCATGTCC-3′ and 5′-AAAAGTCGACCGACCTTGATAGGAGCC-3′ were used for amplifying 5′ flank, and primers 5′-AAAACCCGGGTATGATAGCAGCGCAGCTG-3′ and 5′-AAAAGGATCCCTGCCTCATTGTGCATGTGC-3′ were used for amplifying 3′ flank of the gene. The 5′F and 3′F DNA fragments were cloned on either side (at HindIII/SalI and SmaI/BamHI site) of the neomycin and hygromycin genes of pXG-neo and pXG-hyg vectors, respectively. Both constructs were then digested with HindIII and BamHI to get linear fragments of gene deletion constructs LmGAPDH::NEO and LmGAPDH::HYG and were transfected into *L. major* sequentially as described (56). The knock out strain was cultured in the presence of 50 μg/ml neomycin and 100 μg/ml hygromycin drug.

### Complementation of LmGAPDH in null mutants

To restore LmGAPDH in the HKO parasites, LmGAPDH ORF was PCR amplified using an LmGAPDH-containing plasmid as template and the following forward primer 5′-AAAAGGATCCATGGCTCCTATCAAGGTCGG-3′ and reverse primer 5′-AAAAGGATCCCTACATCTTGGCGCTCGCAG-3. The amplified product was cloned at the BamHI site of pXG-PHLEO vector, and 10 μg of the pXG-PHLEO-LmGAPDH was transfected into the HKO promastigotes. Transfected promastigotes were selected with minimal doses of phleomycin (5 μg/ml) and finally were maintained in the presence of 60 μg/ml neomycin, 100 μg/ml hygromycin, and 10 μg/ml phleomycin drug.

### Isolation of extracellular vesicles from CT, OE, HKO, and CM cell lines

For all EVs-related experiments, EVs-depleted fetal bovine serum (FBS) was used. EVs-depleted FBS was prepared by ultracentrifugation of the FBS used at 110,000*g* for 5 h. For EV isolation, cells were grown in media made from EVs-depleted FBS. The cell culture having 2 × 10^8^ cells was taken and then centrifuged first at 300*g* for 10 min, then at 2000*g* for 15 min, followed by centrifugation at 10,000*g* for 30 min. All centrifugations were done at 4 °C. The supernatant was then filtered through a 0.22-μm filter unit. This supernatant was overlaid on a 30% sucrose cushion and then centrifuged at 100,000*g* for 90 min at 4 °C. After centrifugation, the supernatant just above the sucrose cushion was discarded leaving behind a narrow layer of medium with EVs at the interface. EVs were resuspended with 1× PBS (filtered) and again centrifuged at 100,000*g* for 90 min at 4 °C. The pellet was used as the EVs.

### Isolation of glycosomal fractions

Glycosome was isolated from *L. major* promastigotes using Colasante *et al.*'s (34) technique with slight modifications. *L. major* promastigotes were harvested by centrifugation for 10 min at 2000*g* and were washed once in 10 ml of wash buffer (25 mM Tris, 1 mM EDTA, 1 mM DTT, 250 mM sucrose, pH 7.8). After centrifugation, the cell pellet was resuspended in 2 ml homogenization medium (250 mM sucrose, 1 mM EDTA, 0.1% (v/v) ethanol, 5 mM MOPS, pH 7.2) containing protease inhibitor (complete EDTA-free, Roche Applied Science) and was lysed using syringe (needle gauge-0.30 × 12.5 mm). Cell lysis was confirmed by light microscopy. The cell lysate was centrifuged sequentially for 5 min each at 100*g* and 3000*g* to remove cell debri and cell nuclei. The resulting supernatant was centrifuged at 17,000*g* for 15 min to obtain glycosomal-enriched pellet. This pellet was resuspended in 1.0 ml homogenizing buffer and was loaded on top of a 24-ml linear 20% to 40% (v/v) optiprep gradient (Sigma), mounted on 50% 2.5 ml optiprep cushion and centrifuged at 170,000*g* for 1 h. Nine fractions (1.0 ml each) were collected from the bottom of the tube. They were centrifuged at 30,000*g* to obtain the pellet. The pellet was resuspended in homogenizing buffer and again centrifuged at 30,000*g* to obtain the purified glycosomal pellet. The purity of the fraction was checked by Western blotting using the glycosome-specific rabbit LmPAS-PGK antibody (1:50).

### Subcellular fractionation

Subcellular fractionation was performed to isolate mitochondria, nuclei, and cytosolic fractions using the mitochondria isolation kit (Qiagen). EVs and glycosome were isolated by the method described above. The purity of each fraction was checked by Western blot analysis with organelle-specific marker antibodies. The primary antibodies used were as follows: rabbit anti-(*L. major*) GAPDH antibody (1:100), rabbit anti-(*L. major*) PAS-PGK antibody (1:50), rabbit anti-(*L. major*) ascorbate peroxidase (1:50), rabbit anti-(*L. donovani*)-adenosine kinase (1:50), rabbit anti-goat histone 2B antibody (1:2000), and mice anti-(*L. donovani*) GP63 antibody (1:200). The alkaline phosphatase–conjugated anti-rabbit secondary antibody (Sigma) was used at a dilution 1:15,000.

### *In vitro* promastigote growth profile analysis

A total of 10^6^ mid-log phase cells were inoculated in 10 ml of M199 medium supplemented with 10% FBS. Growth rates were measured at a 24-h interval by counting cell number in an improved Neubauer chamber (hemocytometer) for eight consecutive days. Experiments were done in triplicate for each cell type.

### *In vitro* macrophage infection

Stationary phase CT, OE, CM, and KO promastigotes were used to infect cultures of the adherent murine macrophage cell line RAW264.7 on glass cover slips (22 mm; 5 × 10^5^ macrophages/cover slip) in 0.5 ml of RPMI 1640/10% FBS mixture at a parasite to cell ratio of 10:1. Following the incubation, unphagocytosed parasites were removed by washing with medium, and cells were resuspended in RPMI 1640/10% FCS mixture at 37 °C. A 2-h incubation period was used for the determination of parasite entry. Cultures were transferred to a CO_2_ incubator at 37 °C for an infection period of 24, 48, and 72 h. Cells were then fixed in methanol and stained with propidium iodide. Cells were visualized and quantified using Olympus IX81 microscope.

### Infection in mice

Female BALB/c mice 6 to 7 weeks old were infected subcutaneously with 5 × 10^6^ stationary-phase promastigotes in their left hind footpads (eight mice per group). Disease progression was monitored by daily caliper measurement of footpad swelling. Parasite loads in footpad of mice containing WT, OE, CM, and HKO parasites were determined by limiting dilution assay with slight modification. Footpad tissue were sequentially immersed in 70% ethanol and sterile water before homogenization of weighed tissue in M199 medium supplemented with gentamycin and penicillin–streptomycin containing 10% heat-inactivated FBS. Each tissue homogenate was serially diluted in the same medium in a 96-well flat-bottomed tissue culture plate. The number of viable parasites per milligram of tissue was determined from the highest dilution at which promastigotes could be grown out after up to 10 to 15 days of incubation at 22 °C.

### Real-time PCR for measuring mRNA expression of TNF-α cytokines in parasite-infected macrophage

Real-time PCR was performed to investigate the relative quantities of cytokines TNF-α mRNA. Briefly, RAW264.7 cells (1 × 10^6^ cells/ml) were plated in six-well plates for around 24 h. The plates were then washed with RPMI-1640 and reincubated with LPS (1 μg/ml) or different type of *Leishmania* promastigotes (1:10 ratio) at different time periods. Total RNA was isolated from the parasite-infected RAW264.7 cells using Trizol reagent (Invitrogen Life Technologies) according to the manufacture's protocol. cDNA synthesis was then performed using High Capacity cDNA Reverse Transcription Kit (Applied Biosystems, ABI). Real-time quantitative PCR was performed on the StepOne Real-Time PCR system (Applied Biosystems, ABI) using Power SYBR Green PCR Master Mix (ABI). Relative expression levels of mRNA were normalized using control cells as reference sample and beta-actin as the endogenous control using a comparative ΔΔct method as described by the manufacturer. Primers 5′-GGATTATGGCTCAGGGTCCA-3′ (forward) and 5′-ACATTCGAGGTCCAGTGAA-3′ (reverse) for TNF-α and primers 5′-TTGTGATGGACTCCGGAGAC-3′ (forward) and 5′-TGATGTCACGCACGATTTCC-3′ (reverse) for beta-actin were used.

### Cytokine measurement by ELISA

The cell culture supernatant from the *L. major*/macrophage coculture and EVs-treated macrophage was collected and centrifuged to remove noninternalized parasites and debris. The production of proinflammatory cytokine TNF-α was quantified using TNF-α Quantikine ELISA kits (R&D systems) according to the manufacturer's instructions.

### Cytokine measurement by Western blotting

Cells were collected following infection for 24 h and lysed in lysis buffer (Cell Signaling Technology). The protein concentrations in the cleared supernatants were estimated using a Bio-Rad protein assay mixture. Fifty micrograms of proteins was subjected to 10% SDS-PAGE, and proteins were transferred to a nitrocellulose membrane (Millipore) and blocked for 2 h at 37 °C in TBS-T (20 mM Tris-HCl, 137 mM NaCl, 0.05% (v/v) Tween 20, pH 7.6) containing 5% BSA to prevent nonspecific binding of antibodies. Immunoblotting was performed by incubating in TNF-α monoclonal primary antibody (Abcam) at 1:1000 dilution overnight at 4 °C. Membranes were washed three times in TBS-T, followed by incubation with anti-rabbit IgG coupled to alkaline phosphatase (1:15,000). Again, after three washes with TBS-T, membranes were developed by NBT-BCIP (Roche) method.

### *In vitro* protein translation of TNF-α mRNA in presence or absence of purified leishmanial GAPDH

Total RNA was isolated from the parasite-infected RAW264.7 cells using Trizol reagent (Invitrogen Life Technologies) according to the manufacture's protocol. LPS (20 μg)-induced mRNA expression in Raw 264.7 cells was subjected to *in vitro* translation by addition of 35 μl rabbit reticulocyte lysate (Promega), 20 mM methionine free amino acid mixture, 40 U of RNase inhibitor (Roche), and 20 μCi of ^35^S methionine with or without LmGAPDH in a 50-μl reaction for 90 min at 30 °C. A 45-μl aliquot was subjected to immunoprecipitation with TNF-α monoclonal antibody (Abcam) and protein A/G plus agarose (Santa Cruz Biotechnology) in a buffer containing 50 mM Tris-Cl (pH 7.6), 50 mM NaCl, 1 mM PMSF, 1 mM DTT, 0.5% NP40, 1× protease inhibitor cocktail (Roche). Immunoprecipitated proteins were resolved on 10% SDS-PAGE. To analyze the general translation pattern, a 5-μl aliquot that was not subjected to immunoprecipitation was resolved by 10% SDS-PAGE. The gels were analyzed autoradiographically by fixing, drying, and exposing the gel on X-ray film as mentioned above.

### *In vitro* LmGAPDH protein transfection

Adherent RAW 264.7 cells were grown on six-well plates in RPMI 1640 and 10% FBS. Recombinant LmGAPDH protein was purified according to the protocol mentioned above. Purified protein in a suitable buffer (10 mM Tris, 150 mM NaCl, pH 7.0) was transfected to the adherent RAW cells through Pierce Protein Transfection Reagent kit (Thermo Scientific) following manufacture's instruction.

### Immunofluorescence and confocal microscopy

The transfected cells grown on glass cover slips were washed in 1× PBS and then the cells were fixed by 4% paraformaldehyde. Cells were then permeabilized with 1.0% Triton X 100 in 1× PBS with 50 μg/ml RNaseA (Calbiochem). The cover slips were air dried and blocked with 1.5% BSA for 2 h. The cover slips were then incubated with anti-rabbit GAPDH antibody (1:200) overnight at 4 °C. On the next day the cover slips were washed with 1× PBS and incubated with Alexa fluor 488-conjugated anti-rabbit secondary antibody (Life Technologies) at a dilution of 1:600 for 2 h. Finally, they were mounted on a slide with DAPI-containing antifade mounting media (Invitrogen) and observed under confocal microscope (Leica). The wavelengths used were DAPI, Ex_λ_ = 350 nm and Em_λ_ = 470 nm; Alexa Flour 488 secondary antibody, Ex_λ_ = 488 nm and Em_λ_ = 522 nm.

### RNA electrophoretic mobility shift assay

For our experiment, we used AU-rich RNA derived from TNF-α 3′ UTR (ARE38 5′-GUGAUUAUUUAUUAUUUAUUUAUUAUUUAUUUAUUUAG-3′) and an arbitrary region derived from TNF-α 5′ UTR (5′-AGAACAUCUUGGAAAUAGCUCCCAGAAAAGCAAGCAGC-3′) synthesized and biotin end-labeled by using biotin at 3′ end by Biotech Desk Pvt. Ltd. RNA-EMSA was then performed with the Light Shift chemiluminescent RNA EMSA kit (Thermo Scientific) according to the manufacturer's instructions. For performing RNA binding reactions, purified LmGAPDH protein and 2 nM of biotin-labeled probe in a volume of 20 μl were incubated together for 30 min at room temperature along with 2 μg tRNA, 5% glycerol in 1× of REMSA binding buffer. For monitoring cold competition assays, unlabeled TNF-α 3′ UTR RNA oligonucleotides were added to the reaction mixture (∼200-fold excess) before addition of the labeled probes. The REMSA supershift is carried out using another lane loaded with labeled probe, LmGAPDH purified protein, and LmGAPDH antibody (raised) specific for the protein. Increasing concentrations of NAD+, NADH, and ATP were added to the reaction mixture to ensure their effect on LmGAPDH-mediated inhibition of TNF-α production. Samples were separated on native polyacrylamide gels (6%) in 0.5× Tris-borate-EDTA (TBE) buffer at 100 V at 4 °C that were prerun for 30 min. Then the gel was transferred into positively charged nylon membranes (Sigma) in Trans-Blot Semi dry apparatus (Bio-Rad). The biotin-labeled RNA on the membrane was cross-linked by a UV-light cross-linker (UVP HL-2000 HybriLinkerTM) and then detected using horseradish peroxidase–conjugated streptavidin. Band shifts were observed by exposure using iBright Imaging System (Invitrogen).

### Atomic force microscopy for visualizing extracellular vesicles structure

For AFM imaging, purified EVs preparations were diluted with filtered PBS (1:100) and a 5 μl aliquot of the diluted sample solution was deposited on freshly cleaved mica sheet followed by incubation at room temperature for 10 min. The dried sample was then gently washed with 200 μl of Milli-Q water to remove salt and loosely bound moieties. AFM experiments were performed in intermittent contact mode (called “tapping” or AAC mode) to minimize tip-induced damage. AAC mode AFM was performed using a Pico plus 5500 inverted light microscope AFM (Agilent Technologies) with a Piezo scanner (maximum range 9 μm). Microfabricated silicon cantilevers 225 μm in length with a nominal spring force constant of 21 to 98 N/m were used (Nano Sensors). Cantilever oscillation frequency was tuned into resonance frequency. The cantilever resonance frequency was 150 to 300 kHz. The images (512 × 512 pixels) were captured with a scan size between 0.5 and 0.8 μm at a scan speed rate of 0.5 lines/s. Images were processed by flatten using Pico view1.1 version software (Agilent Technologies). Image manipulation was done using Pico Image Advanced version software (Agilent Technologies).

### Dynamic light scattering Zeta potential measurements

The extracted EVs (100 μl) were diluted 10 times using 1 × PBS and then transferred into a disposable cuvette (40 μl), and then the EVs-containing cuvette was put into the DLS device to start measuring. All size distribution and ζ-potential experiments were conducted using the Nano-ZS instrument (Malvern Instruments) at 25 °C (5 mW, He–Ne laser, λ = 632 nm, scattering angle = 173°). For determining size distributions by DLS, the operation procedure was programmed using the DTS software supplied with the instrument to record the average of an automated number of runs. Every run was collected for 10 s, and an equilibration time of 5 min was used, prior to starting the measurement. For all ζ-potential measurements, samples were loaded into a folded capillary cell and average ζ-potential values obtained from an automated number of runs (maximum 100) were noted. The instrument measured ζ-potential from the electrophoretic mobility (μ), using a model described by the Smoluchowski equation.

### Statistical analysis

All data were expressed as the mean ± SD from at least three independent experiments. Statistical analysis for parametric data was performed by Student's *t* test or analysis of variance (ANOVA) wherever applicable using Origin 7.0 software (Microcal software, Inc). A *p* value of less than 0.05 was considered statistically significant.

## Data availability

All data generated and analyzed during this study are contained within the article.

## Conflict of interest

The authors declare that they have no conflicts of interest with the content of this article.
